# In vivo models of brain tumors: roles of genetically engineered mouse models in understanding tumor biology and use in preclinical studies

**DOI:** 10.1007/s00018-014-1675-3

**Published:** 2014-07-10

**Authors:** Iva Simeonova, Emmanuelle Huillard

**Affiliations:** Université Pierre et Marie Curie (UPMC) UMR-S975, Inserm U1127, CNRS UMR7225, Institut du Cerveau et de la Moelle Epiniere, 47 boulevard de l’Hôpital, 75013 Paris, France

**Keywords:** Mouse models, Glioma, Medulloblastoma, Cell of origin, Neural stem cells

## Abstract

Although our knowledge of the biology of brain tumors has increased tremendously over the past decade, progress in treatment of these deadly diseases remains modest. Developing in vivo models that faithfully mirror human diseases is essential for the validation of new therapeutic approaches. Genetically engineered mouse models (GEMMs) provide elaborate temporally and genetically controlled systems to investigate the cellular origins of brain tumors and gene function in tumorigenesis. Furthermore, they can prove to be valuable tools for testing targeted therapies. In this review, we discuss GEMMs of brain tumors, focusing on gliomas and medulloblastomas. We describe how they provide critical insights into the molecular and cellular events involved in the initiation and maintenance of brain tumors, and illustrate their use in preclinical drug testing.

## Introduction

Primary brain tumors originate from the transformation of neural stem cells (NSC) or cells committed to the neuronal, astrocytic and oligodendrocytic lineages. In adults, gliomas are the most common primary brain tumors, accounting for about 30 % of all primary brain and central nervous system tumors, and 80 % of malignant tumors, according to the Central Brain Tumor Registry of the United States [1]. High-grade gliomas, such as glioblastomas (GBM) and high-grade astrocytomas (HGA) have a poor prognosis due to their resistance to conventional radio- and chemotherapies. In children, medulloblastomas (MB) are the most common brain tumors. Although conventional therapies can cure subsets of MB patients, the treated patients face long-term neurological side effects [2].

The etiology of most brain tumors is not well understood. As we will discuss below, thanks to recent large-scale efforts on the molecular characterization of MB and GBM, it is now clear that these tumors, initially thought as unique entities, comprise distinct diseases at the clinical and molecular levels. The molecular and cellular mechanisms underlying tumor formation and heterogeneity are just starting to emerge. Better understanding of brain tumor development and biology is critical for the identification of new targets and the design of successful therapies.

## What is a good model of brain tumor?

The goal of a model is to reproduce the etiology and biology of the corresponding human disease, to understand its development and to identify adequate treatments. Ideally, a good model should (1) display the same genetic lesions, anatomical location, histopathological features, and developmental time frame as the human tumor; (2) recapitulate intertumoral and intratumoral heterogeneity; (3) be predictive of the patients’ response to treatment.

Cell lines derived from brain tumors are useful for the characterization of the genetic lesions that occur in human tumors, and to build primary hypothesis about gene function. However, they cannot model effectively the key aspects of tumorigenesis, such as microenvironment contribution, invasion, angiogenesis or inflammatory response. Therefore, they only have a limited predictive value for the development of cancer therapies. In vivo models provide a more accurate experimental system, as they mimic tumor behavior in an entire mammalian organism.

## In vivo approaches for modeling brain tumors

Numerous in vivo models of brain tumors have been developed, including carcinogen-induced rodent models, xenograft and genetically engineered mouse models (GEMMs) (Table 1). Rat models have been extensively used since the mid-1970s. Gliomas could be induced in rats injected with the alkylating agents *N*-methylnitrosourea (MNU) or *N*-ethyl-*N*-nitrosourea (ENU). These models have the advantage of developing tumors de novo, preserving tumor–host interactions. However, in many of these models, tumors grow as circumscribed tumors, instead of being invasive like the human tumors [3]. In addition, rat tumors have not been characterized at the molecular level and thus it is not known to what extent their mutational and transcriptional profiles match those of human tumors.

**Table 1:** Overview of the most commonly used models of brain tumors

Model	Host	Advantages	Limitations
In vitro	n/a	Ease of use; minimal cost; readily available	Tumor/host interactions can not be tested
		Large screening possibilities	Drug pharmacological properties can not be addressed
Carcinogen-induced	Rat	De novo tumor formation	Biology and histology different from human tumor (circumscribed tumors)
		Intact immune system	Highly immunogenic (9L cell line)
Xenografts of tumor cell lines (serum conditions)	Immunodeficient mouse	Good reproducibility	Long-term cultures can drift
		Easy to culture and expand	Deficient immune system of the host
		Good engraftment rate	Tumor genomics, transcriptomics and biology different from original tumor
		Monitoring of tumor growth (BLI)	
Xenografts of tumor cell lines (serum-free conditions)	Immunodeficient mouse	Injected cells enriched in brain tumor stem-like cells	Deficient immune system of the host
		Closely mimic genomics and biology of parental tumors	Difficult to establish
		Monitoring of tumor growth (BLI)	
		Short tumor latency	
Genetically engineered mouse models	Immunocompetent mouse	Temporal and spatial control of tumor initiation	Tumor formation in mouse may differ from human
		De novo tumor formation	Important breeding costs
		Intact immune system	Long tumor penetrance and latency
		Monitoring of tumor growth (BLI)	

*BLI* bioluminescence imaging, *n/a* not applicable

Xenograft models are generated by the transplantation of biopsies or cultured cells derived from human brain tumors into immunodeficient mice. Cell lines derived from gliomas and grown in serum-containing medium have been used to develop cytotoxic agents and tumor-specific agents. These cells represent an unlimited source of material for drug testing and engraft easily into immunocompromised mice. However, tumors initiated from cells cultured in serum-containing medium poorly resemble the genotype and phenotype of their parental tumors: they grow as circumscribed, non-invasive tumors and do not display the same transcriptomic profile and genomic alterations as the parental tumors [4–6].

The past decade witnessed the identification, based on the expression of the cell surface marker CD133, of brain tumor stem cells (or brain tumor-initiating cells) in several brain cancers [7, 8]. These cells are able to reconstitute a tumor upon transplantation and display cardinal features of normal NSC, such as the ability to self-renew and to give rise to the three main cell types of the central nervous system (astrocytes, oligodendrocytes, neurons). The cancer stem cell hypothesis postulates a hierarchical tumor organization, where only a small subpopulation of tumor cells drives tumorigenesis. However, this hypothesis is being challenged. Indeed, subsequent studies have shown that CD133-negative cells exhibit similar properties [9, 10]. Actually, GBM tumors have been shown to contain both CD133+ and CD133-negative cell types that generate highly aggressive tumors with different growth kinetics, histology and gene expression profile [9]. These studies suggest that GBMs contain heterogeneous populations of cells with distinct tumor-initiating properties.

By culturing cells freshly derived from GBM patients under NSC serum-free conditions, researchers were able to generate tumors that displayed the biology, genetics and gene expression profiles of the corresponding human GBM [4, 11]. In contrast, tumors generated from cells cultured in serum failed to recapitulate the histopathological features of the parental tumors. In a recent study, Joo et al. [11] established a library of over 50 orthotopic xenografts generated from GBM sphere cultures. They showed that the response of the xenografts to in vivo irradiation and chemotherapy matched the response of the parental GBM [11]. Xenografts of serum-free GBM cultures thus appear to be promising tools for recreating a human brain tumor in the mouse brain. Nevertheless, a limitation of xenograft models is that they usually require injection of a large amount of cells into the host, which does not mirror the formation of human tumors from a restricted number of cells. Moreover, the use of immunodeficient mice does not take into account the contribution of the immune system in tumor development. Some brain tumor types have been more difficult to establish in culture. For example, oligodendroglioma-derived cells are difficult to propagate in vitro or do not engraft in host mice. Only recently, the first oligodendroglioma cell cultures and xenografts bearing typical genetic alterations have been successfully established [12].

## Genetically engineered mouse models of brain tumors

**Fig. 1:**
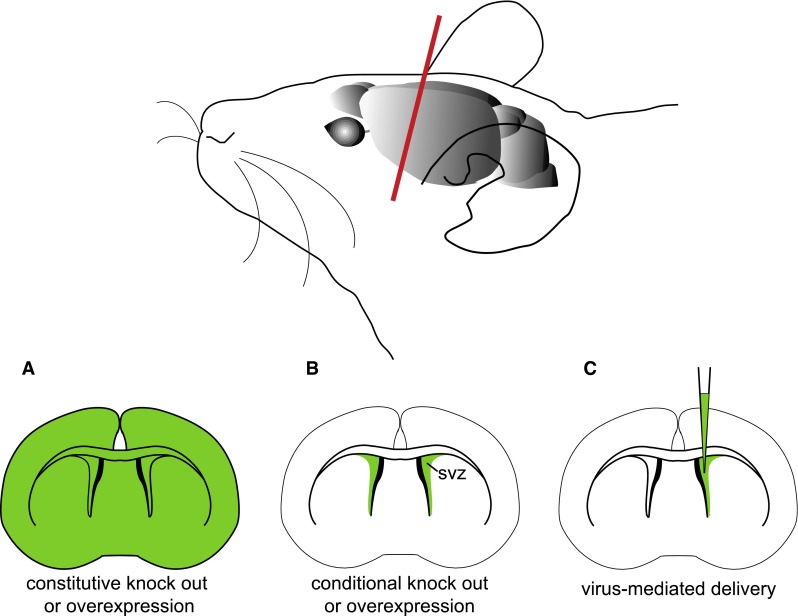
Genetic strategies for the establishment of GEMMs of brain tumors. *Top* a mouse brain is shown with the plan of section in *red*. *Bottom* coronal sections of the mouse brain, with areas of altered expression of gene of interest (GOI) are shown in green. **a** A constitutive knock-out or overexpression targets a GOI in the whole organism. **b** A conditional knock-out or overexpression targets a GOI in a restricted spatial and temporal area, for example in neural stem cells of the subventricular zone (SVZ). **c** Virus-mediated delivery ensures local GOI targeting at the site of injection

The availability of the complete sequence of the mouse genome as well as powerful gene-targeting tools has led to the development of GEMMs to investigate tumorigenesis [13]. In these models, defined gene alterations identified in human tumors (affecting specific oncogenes or tumor suppressor genes) are introduced in the germline (knock-out, knock-in, transgenic models) to allow for de novo tumor formation (Fig. 1a) [14]. Although these models may better mimic tumorigenesis in familial syndromes, deletion of a tumor suppressor gene in the entire organism is likely to lead to a wide spectrum of diseases, including cancers, precluding correct analysis on its implication in brain tumorigenesis. For example, mice constitutively deficient for the tumor suppressor locus *Cdkn2a* (or *Ink4a/Arf*) are viable and fertile but develop fibrosarcomas and lymphomas [15]. As *CDKN2A* loss has been reported in several brain tumors, a specific deletion on the locus in the brain is needed to address its role in this pathology. The development of conditional Cre/loxP systems has enabled to specifically target defined cell populations (Fig. 1b). In this system, the Cre recombinase permits the excision of a gene flanked by two loxP sites. The induction of Cre expression under the control of a tissue-specific promoter allows for targeted deletion in a defined cell type. For example, in the elegant MADM (Mosaic Analysis with Double Markers) model, a mouse genetic mosaic system, it is possible to induce sporadic mutations in a restricted cell population and trace the fate of individual mutated cells and their wild type siblings [16]. This system was used to model high-grade glioma development from NSCs [17]. A Cre that can be induced temporally (Tamoxifen-inducible Cre) further allows to introduce genetic alterations at a given developmental time point, more faithfully modeling somatic tumor development that occur in somatic cells at later stages. Specific cell populations can also be directly modified in a time-dependent manner using virus-mediated gene delivery (Fig. 1c). An example is the RCAS–TVA system which allows to deliver genes of interest by the avian retrovirus RCAS into defined cells engineered to express the RCAS receptor TVA [18]. Transposon-based insertional mutagenesis is another tool for functional mutagenesis and cancer gene discovery in the mouse [19]. Transposable elements (or transposons) are DNA sequences that can change their location in the genome. The transposition system, called Sleeping Beauty (SB), is based on the use of a non-autonomous transposon (T2/Onc) that needs a trans-acting transposase element (SB11) to mobilize and randomly reintegrate elsewhere. The transposon contains a donor splice site and promoter/enhancer sequences, and can deregulate expression of a putative oncogene, when integrated upstream in the same transcriptional orientation. It also contains acceptor splice sites and a bi-directional poly(A) sequence that can terminate transcription of a putative tumor suppressor gene. This allows for random loss or gain of function of genes with easy subsequent identification by the transposon sequence inserted. The SB system is also compatible with Cre/loxP, as the transposase element can be conditionally activated (lox-stop-lox-SB11) to allow for tissue-temporal control of transposon mobilization. This mutagenesis strategy has been recently used to perform high throughput screen for genes and signaling pathways involved in NSC transformation into glioma-initiating cells [20, 21]. The SB system was also shown to generate high-grade astrocytoma in mice, at a low frequency [20, 21]. Furthermore, it is useful for the identification of cooperative mutations, when two or more genes are found mutated with high frequency in the same tumors. SB mutagenesis can also accelerate tumorigenesis on a tumor-prone genetic background. The latter was recently used to generate high penetrance mouse models for medulloblastoma that faithfully recapitulate the human pathology [22, 23].

GEMMs have the advantage of using defined genetic alterations to induce tumor development de novo, in an immunocompetent host. Therefore, the entire tumorigenic process, from the transformation of the cells of origin to the full-blown tumor in the appropriate microenvironmental stroma, can be appreciated. GEMMs have been used to investigate the nature of the cell of origin and initiating alterations, gene interactions in tumorigenesis, and the contribution of the tumor microenvironment. Such issues cannot be addressed with end-stage human tumors or using immunodeficient hosts.

## GEMMs to investigate the cell of origin and tumor heterogeneity

The cell of origin can be defined as the cell type that is initially transformed by genetic alterations to initiate tumor formation. NSCs derive from neuroepithelial cells that line the brain ventricles at early developmental stages. These NSCs then divide asymmetrically to generate neural progenitor cells (NPCs), which in turn give rise to neurons, oligodendrocytes and astrocytes. NSCs persist in the adult, and generate subsets of neurons and oligodendrocytes from the subventricular zone (SVZ) and the dentate gyrus of the hippocampus [24].

Identifying the lineage that is originally transformed provides critical insights into understanding tumor mechanisms and for designing rational therapeutic strategies. As discussed below, recent studies that integrate genomic analysis and in vivo modeling approaches have yielded important insights into the etiology and tumor heterogeneity of medulloblastomas and gliomas.

### Medulloblastomas

Medulloblastomas are the most common pediatric brain tumors that also rarely occur in adults. These tumors affecting the cerebellum are heterogeneous at the clinical level: they can be grouped into different subtypes that display distinct histology, prognosis and demographics [2, 25]. At the molecular level, four MB subgroups have been described. The most homogenous group is the Sonic Hedgehog (SHH) Group 1, which is driven by aberrant SHH signaling pathway. Overexpression of activated *Smoothened* (*SMO*), inactivating mutations of *PTCH1* (*Ptc1* in mice), and *MYCN* amplification, are characteristic for this group. The WNT Group 2 contains activating mutations in *CTNNB1*, and frequent *TP53* (the gene encoding p53) mutations. MB Group 3 and 4 are less well defined, although *MYC* amplification and aberrant expression is frequently observed in Group 3. Group 4 does not display specific alterations; however, duplication of chromosome arm 17q and loss of 17p, called isochromosome 17q (i17q), is observed in over 80 % of the cases [2, 25].

Studies in mouse models have shown that MB subtypes arise from distinct cell populations. In models of Shh pathway-induced Group 1 MB, tumors are generated from Olig2+, GFAP+ or Math-1+ cells, which are related to several stages of cerebellar granule neuron precursors (GNP) development [26, 27]. Shh activation in a distinct Nestin+ quiescent progenitor population also committed to the GNP lineage exhibited even more aggressive tumorigenesis, due to intrinsically increased genomic instability [28]. Conversely, no tumors formed when a non-granule cerebellar lineage, such as Purkinje cells, is targeted [26, 27]. In striking contrast, a mouse model of WNT Group 2 MB that overexpresses an activated form of *Ctnnb1* in hindbrain neural progenitors, develop tumors from the dorsal brainstem, and not from the cerebellum [29]. These mouse models faithfully recapitulate human MB tumors, in which SHH- and WNT-subtypes tend to be also anatomically distinct [29]. Indeed, nearly half of SHH-subtype tumors are located within the cerebellar hemispheres, whereas WNT-subtype, locate close to the dorsal surface of the brainstem. The evidence of heterogeneity within subgroups adds an additional level of complexity. For instance, Group 1 SHH-driven MB is most prevalent in infants and adults, yet these tumors have distinct transcriptional profiles [2]. Mouse models for Myc activation Group 3 MB, the most aggressive type of MB, have recently been reported [30, 31]. In these models, orthotopic transplantation of GNP cells or cerebellar NSCs expressing Myc and mutant p53 leads to the formation of MB that bear histological and transcriptional features of human Group 3 tumors. In addition, the expression profiles of the mouse tumors matched those of NSCs, suggesting that Group 3 tumors arise from NSCs or de-dedifferentiated GNP cells upon Myc expression (Table 2).

**Table 2:** Human tumor subgroups and proposed cells of origin based on GEMM studies

Corresponding human brain tumor	Targeted cell population	Cre driver	Initiating alterations	Phenotype	Frequency/latency	Main contribution of the model	Limitations	References
Medulloblastomas								
Group 1 SHH pathway	Granule neuron precursors (cerebellum)	hGFAP-Cre, Olig2-tva-cre, Math1-Cre	**SmoM2**	Medulloblastoma	100 %/1–2 months	Acquisition of granule cell lineage identity is required for Shh-driven MB formation	Transcriptional match with human Group 1 MB has yet to be demonstrated	[26]
	Granule neuron precursors (cerebellum)	Math1-CreER	*Ptc*	Medulloblastoma	100 %/2–3 months	Shh pathway activation in stem cells promotes stem cell proliferation but only causes tumors after commitment to, and expansion of, the granule cell lineage	Transcriptional match with human Group 1 MB has yet to be demonstrated	[27]
Group 2 WNT pathway	NSC (hindbrain)	BLBP-Cre	**Ctnnb1,** *p53*	Medulloblastoma, WNT- subtype	20 %/6–9 months	Progenitor cells within the dorsal brainstem are susceptible to transformation by concurrent mutation in Ctnnb1 and p53, resulting in the formation of tumors that mimic the anatomical features of human WNT-subtype medulloblastoma	Low penetrance and long latency	[29]
Group 3 Myc activation	Granule neuron precursors (cerebellum)	N/A	*Cdkn2c, p53,* **Myc**	Medulloblastoma, MYC subtype	NR/1 month	This models mimics human MYC subgroup of MB and significantly differs from mouse models of the Shh and WNT subgroups	Tumors do not arise de novo (orthotopic transplantation model)	[30]
	NSC (cerebellum)	N/A	*p53,* **Myc**	Medulloblastoma, MYC subtype	NR/1.5–3 months	Stem cells and GNPs can both serve as cells of origin for MYC-driven MB, suggesting that lineage commitment is not required for transformation	Tumors do not arise de novo (orthotopic transplantation model)	[31]
Group 4	Unknown		ND					
Gliomas								
High-grade astrocytoma or Glioblastoma								
ND	NSC	GFAP-Cre	*p53, Nf1*	Grade II to IV astrocytoma	100 %/5–10 months	Presymptomatic lesions reside within the subventricular zone (SVZ)	Cre active not only in adult NSC but also embryonically, in both astrocytes and NSC; long latency	[52]
		GFAP-Cre	*p53, Nf1, Pten*	High-grade astrocytoma	100 %/5–8 months	Heterozygosity of Pten causes accelerate tumor formation		[51, 126]
	Adult NSC	Adenovirus-Cre	*p53, Pten*	Low to high grade astrocytoma	30 %/7–8 months	Neural stem cells, but not astrocytes, give rise to brain tumors	Low penetrance	[54]
		Nestin-CreERT2, Adenovirus-Cre	*p53, Nf1, Pten*	High-grade astrocytoma	100% / 4–11 months	Adult neural stem/progenitor cells can give rise to malignant astrocytomas. Targeting non neurogenic regions does not induce tumors	Mice injected at 4 weeks of age have a long latency of tumor formation (11 months)	[53]
Proneural	Oligodendrocte precursor cells (subcortical white matter)	Retrovirus-Cre	*p53, Pten,* **PDGF**	GBM	100 %/1 month	Glial progenitors in the adult subcortical white matter can give rise to brain tumors; tumors resemble human proneural GBM and express signatures of OPCs	Retroviral infection may target dividing cells other than OPCs	[56]
	Oligodendrocyte precursor cells	NG2-Cre	*p53, Nf1*	High-grade astrocytoma	86 %/7–8 months	OPCs as the cell of origin in this model, even when initial mutations occur in NSCs. Aberrant growth of OPCs, but not NSC, in premalignant stages	Cre driver is active in both embryonic and adult cells	[17]
	NSC	Nestin-Cre or hGFAP-Cre	*p53, Nf1*	High-grade astrocytoma	77–93 %/4–5 months			
	Adult NSC or astrocytes	GFAP-CreER	*p53, Pten, Rb1*	High-grade astrocytoma, proneural phentoype	NR/5 months	22% of tumors develop from non-proliferative regions. Extensive analysis of molecular (genomic, transcriptomic, RTK expression) characteristics of the tumors shows that several subgroups are generated from the same initiating alterations	Although Cre is active only in mature brain, its expression is not specific to NSC (also active in parenchymal astrocytes). Difficulty to establish the correlation cell of origin/tumor subgroup phenotype	[58]
Mesenchymal	Adult NSC or astrocytes from pons and basal hypothalamus	GFAP-CreER	*p53, Pten, Rb1*	High-grade astrocytoma, mesenchymal phenotype	NR/5 months	NSC and/or astrocytes from the pons and basal hypothalamus are molecularly distinct from NSC and astrocytes in other brain regions		
	Mature neurons (cortex)	SynI-Cre	*p53, Nf1*	GBM	100 %/1.5–2.5 months	Mature neurons can generate high grade gliomas	Dedifferentiation of mature cell types may occur in a minority of GBM in humans	[57]
	Mature astrocytes (cortex)	GFAP-Cre	*p53,* **HRasV12**	GBM, mesenchymal phenotype	100 %/1.5–2.5 months	Mature astrocytes can generate high grade gliomas		
	Adult NSC and astrocytes	hGFAP-CreERT2	*Rb/p107/p130*, **KRasG12D** *, Pten*	GBM	90 %/2 months	This model reconstitutes the sequence of events necessary for GBM tumor initiation and progression: loss of Rb initiates gliomagenesis, KRas signaling drives tumor progression, and loss of Pten drives progression to grade IV	Can not distinguish between NSC or astrocyte cell of origin (both express GFAP)	[59]
Neural	NSC/NPC	GFAP-Cre, Nestin-Cre, Sox2-Cre	*p53,* **HRasV12**	GBM, neural phenotype	100 %/1.5–2.5 months	Targeting mature astrocytes generate tumors similar to the mesenchymal subgroup of GBM, while targeting NSCs with the same mutations result in tumors with a neural signature	Cre driver is active in both embryonic and adult cells	[57]
Oligodendroglioma	Oligodendrocyte precursor cells	S100b-Cre	*p53,* **EGFR**	High-grade oligodendroglioma and GBM	23–46 %/2 months	Oligodendroglioma cells show hallmarks of OPCs rather than NSCs, suggesting an OPC origin for oligodendroglioma	Low penetrance. Not a pure oligodendroglioma model	[55, 82]
Pilocytic astrocytoma	NSC (third ventricle)	GFAP-Cre, GFAP-CreER	*Nf1*	Optic nerve glioma	100 %/8 months	Third ventricle NSCs are molecularly and functionally distinct from SVZ NSC and are the likely cell of origin for low-grade optic gliomas. Embryonic, but not postnatal Nf1 Inactivation Is Required for Optic Glioma Formation	Can not distinguish between NSC or astrocyte cell of origin (both express GFAP)	[40, 99]
Supratentorial ependymoma (subgroup D)	Embryonic NSC (cerebrum)	N/A	*cdkn2a,* **Ephb2**	Ependymoma	50 %/6–7 months	Specific combination of embryonic cerebral NSCs, deletion of cdkn2a and amplification of EphB2 generates supratentorial ependymoma, No tumors form when Ephb2 signaling is activated in adult cerebral or spinal Cdkn2a-/- NSCs	Tumors do not arise de novo (orthotopic transplantation model)	[36]

Bold refers to activated/overexpressed genes, whereas italics refers to gene loss/inactivation

A GEMM using alterations typical of the classic GBM subgroup has been described [125]; however, whether the tumors generated match the classical subgroup at the transcriptional level has not been assessed

*NSC* neural stem cells, *NPC* neural progenitor cells, *HGA* high-grade astrocytoma, *ND* not determined, *NR* not reported, *N/A* not applicable

Taken together, these data suggest that subgroups of MB may represent different diseases with distinct origins and distinct driver mutations, which have implications for the efficacy of targeted therapies (see discussion below).

### Gliomas

Gliomas are tumors that are named after the glial cell type they show morphological similarities with. For example oligodendrogliomas display features of oligodendrocytes, whereas astrocytomas display features of astrocytes. The most common glioma types include ependymomas, astrocytomas, oligodendrogliomas, and mixed gliomas.

#### Ependymomas

Ependymomas (EP) represent only 7 % of all gliomas [1] yet are the third most frequent primary brain tumor in children [32]. These tumors display morphological characteristics of ependymal cells, the cells lining the ventricles of the brain and the spinal canal. Tumors can arise from different regions along the neuraxis: cerebral hemispheres (supratentorial EP), cerebellum (posterior fossa EP) and spinal cord (spinal EP). Although ependymomas are histologically similar, they have disparate prognosis, gene expression and genetic alterations profiles [33, 34]. In 2005, the group of R. Gilbertson revealed that human supratentorial and spinal EP, which are histologically identical, exhibit gene expression profiles similar to murine NSCs of the cerebral ventricle and spinal cord, respectively [35]. In 2010, the same authors identified molecular subgroups of ependymomas, characterized by distinct chromosomal alterations, focal amplifications and deletions [36]. Importantly, they identified previously unknown potential ependymoma oncogenes, such as *EPHB2*, which is selectively amplified and overexpressed in the supratentorial subgroup of EP. By comparing the transcriptome of EP from different regions with that of mouse NSCs of different location and developmental stage, further showed that subgroups of ependymoma match regionally, developmentally and genetically distinct NSCs. For example, the transcriptome of a subgroup of supratentorial ependymoma with amplified *EPHB2* and *CDKN2A* loss (subgroup D) closely matched that of embryonic cerebral *Cdkn2a* ^−*/*−^ NSCs, whereas spinal ependymomas resembled adult wild-type NSCs from the spinal cord. Activation of Ephb2 signaling in embryonic cerebral *Cdkn2a* ^−*/*−^ NSCs generated tumors when these cells were transplanted into immunodeficient hosts. This became the first model of EP, accurately recapitulating the tumor histology and gene expression profiles of the EPHB2/CDKN2A subgroup. It also revealed high enrichment for regulators of neural differentiation and maintenance, particularly ion transport and synaptogenesis, suggesting a previously unsuspected role for this pathway in this particular ependymoma subgroup formation [36]. Contrary to embryonic cerebral NSC, no tumors were generated when Ephb2 signaling was activated in adult cerebral or spinal *Cdkn2a* ^−*/*−^ NSCs [36]. Interestingly, supratentorial tumors tend to occur more frequently in children, whereas spinal EP mostly occurs in adults. These results suggest that supratentorial EP may derive from the transformation of NSCs during embryonic development, whereas spinal EP may originate from the transformation of NSCs during adulthood. Models for other ependymal subgroups, for instance adult spinal ependymoma, remain to be established.

#### Pediatric astrocytomas

The molecular alterations underlying the development of astrocytomas in children are not as characterized as in adults. Molecular alterations in high-grade pediatric astrocytomas (anaplastic astrocytoma and GBM) are starting to be described [37, 38], but in the case of low-grade tumors, the cellular origins and molecular alterations are less well understood. Pilocytic astrocytomas (PA) are low-grade tumors that often develop in the optic pathway and cerebellum, and are frequently observed in patients affected with neurofibromatosis type 1 (NF1) genetic disorder [39]. In a mouse model of pediatric astrocytoma deficient for the *Nf1* gene, Lee da et al. [40] recently showed that for optic glioma to develop, *Nf1* loss must occur in a restricted cell population within a restricted developmental window. Indeed, inactivation of *Nf1* in embryonic NSCs from the third ventricle resulted in optic glioma formation, but inactivation in adult NSCs failed to trigger gliomagenesis. Interestingly, only NSCs from the 3rd ventricle (and not those of the lateral ventricle) are sensitive to specific mutations of PA, such as *KIAA1549:BRAF*, a gene fusion found in the majority of PA located in the hypothalamus/optic pathway regions [40, 41]. In accordance with this data, third ventricle NSCs are molecularly distinct from NSCs of the subventricular zone, the latter being the supposed cells of origin for adult gliomas [40]. Together, these observations point NSC from the third ventricle as putative cells of origin for pediatric optic gliomas.

#### High-grade astrocytomas and glioblastomas

Glioblastomas (GBM or high-grade astrocytomas WHO grade IV) represent 54 % of all gliomas [1]. Most GBM in the adult arise de novo, in the absence of a preexisting tumor (primary GBM). Secondary GBM progress from a low-grade astrocytoma, and occur in younger patients. In 2008, the Cancer Genome Atlas Research Network reported a comprehensive genomic and transcriptomic characterization of over 200 GBM cases. This study showed that the vast majority of GBM harbor alterations in three core signaling pathways: receptor tyrosine kinase (RTK)/Ras/phosphoinositide 3-kinase (PI3K), p53 and RB pathways [42]. Subsequent studies have classified human GBM into several subgroups (classical, mesenchymal, neural, proneural), based on their genomic alterations, gene expression and DNA methylation profiles [43–46]. Correlations with clinical data revealed that the proneural subgroup is associated with better survival, whereas the mesenchymal subgroup has the worst prognosis and is more resistant to conventional therapies. Recently, Sturm et al. [47] have further refined this classification by subclassifying GBM into six groups, with respect to characteristics in global DNA methylation and transcriptome patterns, hotspot mutations, DNA copy-number alterations, patient age and tumor location.

The majority of GEMMs of GBM have used combinations of tumor suppressors *p53* and/or *Rb* inactivation (directly or through *Cdkn2a* deletion), and the activation of pro-survival RTK and Ras signaling (through *Pten* and *Nf1* deletion or RTK/Ras activation) [6, 48–50]. These models have provided essential clues on the identity of cell of origin for GBM and HGA. The group of L. Parada has developed a series of mouse strains harboring conditional alleles for *Nf1*, *p53* and *Pten* [51, 52]. These models demonstrated that adult neural stem/progenitor cells can give rise to malignant astrocytomas in vivo, whereas more mature cell types cannot [53]. This finding was confirmed in a different model developed by Jacques et al. [54]. The authors used adenovirus-mediated Cre delivery to delete *Pten* and *p53* in adult NSCs of the subventricular zone and in mature parenchymal astrocytes. They found that specific deletion of both genes in adult NSCs, but not astrocytes, gave rise to brain tumors. Other studies suggest that oligodendrocyte precursor cells (OPCs) can serve as tumor-initiating cells [17, 55, 56]. Liu et al. used the MADM system to generate high-grade astrocytomas by initiating *p53* and *Nf1 *deletion specifically in NSCs. Interestingly, they found that the population that massively expanded at premalignant stages was OPC —not NSCs or other lineages— and the resulting tumors displayed many features of this cell type. Moreover, introducing the *p53* and *Nf1 *deletion directly in the OPC population resulted in the formation of gliomas indistinguishable from NSC-initiated tumors [17]. These studies suggest a model in which NSCs may be the cells in which the genetic alterations initially occur, but oligodendrocyte progenitor cells may be the glioma-initiating cells of origin, in which the genetic alterations have a functional impact. In contrast with this hypothesis, the Verma group was able to generate high-grade gliomas from the transformation of mature neurons and astrocytes [57]. Lentivirus-mediated knock-down of both *p53* and* Nf1* in mature neurons of the cortex led to the formation of gliomas with GBM features. Likewise, targeting of cortical mature astrocytes with an activated form of Ras (H-RasV12) combined with an shRNA against *p53* induced tumor formation. As tumors progressed, the transduced cells eventually lost expression of the astrocytic marker GFAP and turned on progenitor/stem cell markers. These findings suggest that differentiated cells, by undergoing dedifferentiation or trans-differentiation, can also be the cells of origin for gliomas. Although it is possible experimentally to induce gliomas from differentiated cell types, this mechanism may concern a minority of tumors. Indeed, it seems more likely that NSCs, which cycle throughout the lifespan of the individual, may be more prone to acquire mutations leading to tumor formation.

By comparing the gene expression profiles of GBM subgroups to different murine neural cell types, Verhaak et al. [43, 45] showed that subgroups harbor distinct predominant alterations and match distinct neural cell types. These data suggest that GBM subgroups may arise from distinct cell populations, which are susceptible to distinct genetic alterations. Several GEMMs have been used to test whether combinations of different genetic alterations with different cells of origin generate the different subgroups (Table 2). Knocking-down *p53* and activating Ras signaling in mature astrocytes generated tumors that resembled the mesenchymal subgroup of GBM at the transcriptomic level, while targeting NSCs with the same mutations yielded tumors with a neural signature [57]. In a different model, targeting p53 and Nf1 mutations to OPCs led to tumors that only resembled the proneural subtype of human GBM [17]. Finally, inactivating p53, Pten and Rb1 alleles in both adult NSCs and mature astrocytes generated HGA that could be segregated into three distinct subgroups resembling human molecular subgroups of GBM [58]. These results suggest that subgroups do not necessarily correlate with the nature of the initiating alterations, but rather depend on the identity of the targeted cell type. Interestingly, Chow et al. [58] found that most tumors with mesenchymal subgroup gene signature arose from the pons or the basal hypothalamus. This implies that adult NSCs and astrocytes from these regions are distinct at the molecular level from NSCs and astrocytes from other brain regions. Alternatively, these studies suggest that the regional microenvironment may influence NSC to generate a given subgroup (see section on microenvironment below).

As GEMMs become more sophisticated, they start to provide insights into the different stages of gliomagenesis. By comparing tumor formation in mice inactivated for Rb family members, constitutively activated K-Ras (KRasG12D), Pten loss or combinations of these alterations, Song et al. [59] were able to reconstitute the sequence of events necessary for GBM tumor initiation and progression. Inactivation of Rb family proteins was required to initiate tumorigenesis, and activation of K-Ras signaling induced tumor progression from low-grade to high-grade. This transition was accompanied by the spontaneous occurrence of *p53* mutations. Additional *Pten* loss (engineered or spontaneous) drove progression to grade IV tumors [59].

#### Oligodendrogliomas

Oligodendrogliomas are the second most frequent gliomas in population [60]. Several genomic alterations are commonly found in oligodendrogliomas [61, 62] and frequently associated, indicating that they may play a key role in the initiation and/or maintenance of oligodendrogliomas [63]. The most frequent alteration is a combined loss of one copy of chromosome arms 1p and 19q (1p19q codeletion), which has been observed in 60–90 % of oligodendrogliomas and is associated with a better outcome for patients [61]. Although this alteration was discovered nearly 20 years ago, very little is known on its functional impact on the biology of the tumor. Another common alteration, a heterozygote mutation of *isocitrate dehydrogenase 1* (*IDH1*) on Arg132 (R132H), is found in about 80 % of oligodendrogliomas [64]. The mutated IDH enzyme reduces α-ketoglutarate (α-KG) to D-2-hydroxyglutarate (D-2HG) [65]. D-2HG acts as an oncometabolite, leading to profound cell modifications including histone and DNA hypermethylation, inhibition of cell differentiation and increased proliferation [66, 67]. Interestingly, virtually all 1p19q codeleted gliomas are mutated for *IDH1* [68]. More recently, mutations within the core promoter of *telomerase reverse transcriptase* (*TERT*) have been found in 100 % of oligodendrogliomas with 1p19q codeletion and exclusively in this group of tumors [69]. These mutations confer increased transcriptional activity from the *TERT* promoter [70], and ultimately an increased telomerase activity, which is an important step in the immortalization process [71]. Finally, two recent whole exome studies have reported that the *Capicua transcriptional repressor* gene (*CIC*) is frequently and specifically mutated in 1p19q codeleted oligodendrogliomas [72, 73]. In addition to these genomic alterations, increased EGFR and PDGF/PDGFR expression is frequently observed in oligodendrogliomas [74, 75]. Subgroups are being defined based on these molecular alterations and appear to have distinct prognosis and response to chemotherapy (reviewed in [76]). However, the precise sequence of molecular alterations leading to the oligodendrogliomas formation is not completely understood.

Only a few models for oligodendrogliomas have been developed. These models nonetheless gave critical insights into the nature of the cell of origin for oligodendrogliomas. In most models, activation of the PDGF signaling pathway was used to generate oligodendrogliomas. Retroviral mediated delivery of PDGF-B, a ligand for PDGFRα, in NPCs or OPCs of mouse embryos or neonates induced oligodendroglioma formation [77–79]. Adult progenitor cells seem to require additional alterations to be transformed: infusion of PDGF-B in the lateral ventricles of the adult mouse brain is not sufficient to promote tumorigenesis [80]. Likewise, infection of subcortical white matter OPCs with a PDGF-B retrovirus does not lead to tumor formation, and tumors are generated only in a *Pten*-null; *p53*-null background [56]. However, these tumors resemble GBM and not oligodendrogliomas. Therefore, the nature of the genetic alterations and/or the developmental stage of the targeted cells may determine the phenotype—oligodendroglioma vs GBM—of the tumor. Another model of oligodendroglioma is the GEMM expressing an activated allele of EGFR (v-erbB). Transgenic mice expressing v-erbB in glial cells develop oligodendrogliomas [81]. *v-erbB* mice carrying deletion of *Cdkn2a* or *p53* develop tumors with an increased penetrance and grade. In the *v-erbB;p53*-null model, tumor cells show characteristics of OPCs, similar to human oligodendrogliomas, and it was shown that cells with features of OPCs, rather than NSCs, drive oligodendroglioma formation in mice [55, 81, 82]. In accordance with these data, human oligodendrogliomas were reported to associate with white matter tracts, where OPCs reside, rather than lateral ventricles, suggesting an origin from white matter progenitor cells [55]. Taken together, these studies suggest that OPCs may be the cells of origin for oligodendrogliomas. A GEMM for the 1p19q codeletion has yet to be developed. Furthermore, the recent identification of *IDH1*, *CIC* and *TERT* mutations will lead to new models that should give more insights into the mechanisms of oligodendroglioma genesis.

All together, studies using GEMMs of brain tumors suggest that molecular heterogeneity may be due to subgroups originating from distinct cell types in different brain locations. In addition, populations of neural progenitors appear to be susceptible to particular genetic lesions, suggesting a synergistic effect between these alterations and signaling pathways specific of the cell type of origin [25]. The final goal being the development of effective therapies, a better understanding of the molecular alterations and the role of the microenvironment can lead to the identification of relevant therapeutical targets and to understanding of the mechanisms of therapy resistance.

## GEMMs to investigate interactions between genetic alterations and lineage-specific factors

For their growth, tumor cells rely on cell-specific factors that are normal regulators of the lineage of origin [83]. Tumor growth is driven by cancer stem cells, which share features with normal adult stem cells, such as the ability to self-renew and the potential to differentiate into distinct lineages. Therefore, genes controlling the proliferation and differentiation of normal stem cells may also regulate the biology of cancer stem cells. Mouse models have provided insights into the role of these developmental genes in the tumorigenic process.

Olig2 is a transcriptional repressor that is a key regulator of glial cell fate during CNS development [84]. Olig2 is exclusively expressed in the central nervous system, where it plays distinct roles depending on the developmental stage. Early during CNS development, Olig2 controls the replication-competent state of neural progenitor cells. Later on, it is required for the specification of oligodendrocytes and subsets of neurons [85]. Olig2 protein is expressed in almost all gliomas [86]. Importantly, OLIG2 is expressed in virtually all CD133+ tumor-initiating cells and in the vast majority of Ki67+ proliferating cells in GBM [87], suggesting that it may promote tumor formation. To test this hypothesis, we used an orthotopic mouse model of high-grade astrocytoma that combines loss of the Cdkn2a tumor suppressor and expression of a constitutively activated form of EGFR (EGFRvIII). We found that no tumors formed when Olig2 was absent. We further showed that Olig2 is required for proliferation of tumorigenic neural progenitors, as well as normal progenitor cells. This action is partly mediated through repression of the cell cycle inhibitor p21, an effector of the p53 pathway [87]. We also found that Olig2 affects a key posttranslational modification of p53 in both normal and malignant neural progenitors, thereby antagonizing the interaction of p53 with promoter elements of multiple target genes [88]. Interestingly, Olig2 tumorigenic potential and antagonistic action on p53 is dependent upon its phosphorylation on a triple serine motif: absence of phosphorylation in this region impairs Olig2 pro-tumorigenic activity [89]. These studies identify Olig2 as a regulator of p53 activity in the central nervous system and suggest that Olig2 may contribute to p53 inactivation in the subset of GBM with wild-type p53.

Atoh1 (or Math1) is a proneural basic helix-loop-helix (bHLH) transcription factor highly expressed in GNP cells of the cerebellum [90]. Atoh1 is a key factor in cerebellar development, acting downstream of Shh signaling to regulate GNP proliferation [91]. Importantly, Atoh1 is highly expressed in the Shh-dependent MB subset [92], suggesting that it may act as a lineage dependency transcription factor in these tumors. Indeed, studies using GEMMs have shown that Atoh1 is required for MB formation [91, 93]. Recent work by Forget et al. [94] now provide insights into the mechanisms by which Shh regulate Atoh1 function. Using *Huwe1*-deficient mice, the authors show that Shh regulates Atoh1 stability by preventing its phospho-dependent degradation by the E3 ubiquitin ligase Huwe1 [94]. Atoh1 accumulate in Huwe1-deficient GNPs, leading to migration and differentiation defects. Importantly, Huwe1 is strongly down regulated in tumor-prone *Ptch1* ^+*/*−^ heterozygous mice, and low *HUWE1* expression is associated with poor prognosis only within the SHH subgroup of human MB [94]. This study identifies the developmental Huwe1-Atoh1 module as a critical regulator of SHH-subtype MB.

## GEMMs to study tumor–stroma interactions

The tumor microenvironment represents the non-neoplastic cell types that are embedded in or adjacent to the tumor. It is composed of numerous cell types and molecules, among which immune cells (microglia, the resident immune cells of the brain, peripheral macrophages, infiltrating lymphocytes), extracellular matrix (ECM) components, non-neoplastic neural cells (astrocytes, oligodendrocytes, neurons) and the specialized vasculature structure known as the blood brain barrier (BBB), which is composed of endothelial cells, pericytes, astrocytes). All these cell populations interact with tumor cells to modulate, positively or negatively, tumor growth. GEMMs have provided insights into the contribution of stromal elements, such as microglia and non-neoplastic NPCs, in brain tumor development.

### Microglial contribution to tumorigenesis

Glioma-infiltrating macrophages and microglia constitute a large proportion of tumor mass [95]. Current evidence based on rodent experimental models indicates pro-tumorigenic action of microglia on tumor cells. For instance, GEMMs engineered to express the Herpes Simplex Virus TK specifically in the C11b + microglia/macrophage lineage transplanted with glioma cells and infused with ganciclovir led to an 80 % decrease in tumor volume [96]. Soluble factors released from glioma stimulate microglial toll-like receptors TLRs, resulting in microglial MT1-MMP expression via the TLR downstream signaling molecules MyD88 and p38 MAPK. In turn, MT1-MMP expression and activity in these immune cells promotes glioma cell invasion and tumor expansion [96]. Additional work has shown that microglia may promote glioma cell migration and invasiveness through release of interleukin (IL)-6, IL-18 [97, 98].

Another demonstration of the contribution of microglia to tumor formation came from studies using the *Nf1* GEMM. Mice heterozygous for *Nf1* (*Nf1* ^+*/*−^ mice) or lacking *Nf1* expression in astroglial cells alone (*GFAPCre; Nf1* ^*flox/flox*^ mice) do not develop brain tumors. *Nf1* ± mice with conditional *Nf1* inactivation in astroglial progenitors (*GFAPCre; Nf1* ^*flox/*−^ mice) develop low-grade optic nerve gliomas, similar to children affected with NF1 syndrome [99]. This study shows that non-neoplastic *Nf1* ^+*/*−^ cells provide a permissive environment required for glioma formation. In fact, subsequent studies have revealed that microglia was an important contributor to tumor growth in the *Nf1* model [100]. Genetic ablation of microglia, using a *CD11b-TK* transgenic mouse reduced *Nf1* optic glioma proliferation during both tumor maintenance and tumor development [100]. *Nf1* ^+*/*−^ microglia express high levels of meningioma-expressed antigen-5 (MGEA5) and CXCL12, and these were shown to act as glioma-promoting molecules [101].

Recent studies highlight the importance of microglia in defining tumor subgroups. Indeed, gliomasphere cultures of the proneural subgroup can differentiate into a mesenchymal subgroup. This transition is dependent upon activation of the TNF-α/NK-κB pathway and is accompanied by increased resistance to radiation. Interestingly, TNF-α is secreted by microglia [102]. Microglia has been shown to promote glioma migration and tumor growth and to predominantly infiltrate highly malignant tumors [95]. These observations suggest a role of microglia in a proneural to mesenchymal transition. Importantly, GBM patients with a mesenchymal gene signature and NF-κB activation show a poor response to radiation therapy and have a shorter survival. In this regard, GEMMs will be useful to further explore the influence of regional microglia in mesenchymal GBM development and progression.

### Role of endogenous neural progenitor cells and astrocytes

Mouse models have shown that endogenous NPCs are recruited to the tumor site, where they exert tumor-suppressive activities [95]. GEMMs were used to label specifically NPCs, either through retroviral injection, which labels dividing cells, or through the use of transgenic mice with reporter-gene activity in endogenous NPCs (i.e. Nestin-GFP mouse) by injection of retrovirus [103–105]. In a syngenic glioma model, Walzlein et al. demonstrated that endogenous NPCs engineered to express GFP are recruited from the SVZ to the tumor, where they induce cell death. The same group later demonstrated that NPCs secrete endovanilloids that activate the TRPV1 receptor expressed by glioma cells, triggering the ATF3-dependent ER stress pathway resulting in cell death [106]. Importantly, an endovanilloid agonist was effective against xenografted human GBM cells and prolonged survival [106].

The group of Eric Holland has taken advantage of its PDGF-driven GEMM to characterize non-neoplastic astrocytes in the glioma microenvironment. By combining this model to a GFAP-GFP mouse line to label reactive astrocytes, they showed that tumor-associated astrocytes have increased expression of MHC class II molecules and components of antigen presentation pathway [107]. They identified a gene signature for glioblastoma-associated astrocytes; these genes were mainly expressed in the stromal compartment of the tumor, and were associated with survival in the proneural subtype of human glioma [107].

GEMMs have also been used to show that non-cell-of-origin derived cells within glioma environment in the mouse can be corrupted to become bona fide tumor cells. In a PDGF-driven rat glioma model, Assanah et al. [103] showed that injection of a retrovirus encoding PDGF and GFP induced tumors composed of both GFP+ and GFP-negative cells. In this model, most of the proliferating Ki67+ cells were GFP-negative. Using a PDGF-driven GEMM, Fomchenko et al., also showed that tumors were composed of GFP+ and GFP-negative cells, comprising Olig2+ proliferating NPCs and displaying a gene expression profile similar to that of tumor cells. GFP-negative « recruited » cells were able, upon retransplantation in mice, to initiate gliomas [105]. Whether this applies to human GBM (or GBM of other subgroups) is unknown, as it is almost impossible to distinguish GBM cells from recruited progenitor cells in human tumors, because of their phenotypic similarities. Nonetheless these studies will have to be repeated in other GEMMs and xenograft models.

### GEMMs to study the blood–tumor barrier (BTB)

The blood brain barrier is a specialized vascular structure tightly regulating homeostasis of the central nervous system. It is composed of specialized endothelial cells connected by tight junctions, a capillary basement membrane, astrocyte end-feet ensheathing the vessels, and pericytes [108]. The BBB tightly regulates the influx/efflux of nutrients, endogenous compounds such as hormones and immune cells between systemic circulation and the brain parenchyma. Complex interplay between endothelial cells, ECM, tight junction and astrocyte polarity has to be precisely controlled for barrier integrity. However, the mechanisms involved between the different cell types, as well as their respective role on BBB integrity, are not known. GEMMs have been used to define the contribution of tight junction proteins, transporters, or ECM components in BBB development and biology [109]. GEMMs have been used to demonstrate the role of key signaling, such as VEGF, Notch, Wnt pathways, in BBB development and maintenance. Shh signaling was recently shown to have a protective role at the BBB [110]. Shh is secreted by astrocytes and its receptor Ptc1 is expressed on endothelial cells. Blocking the Hh pathway with cyclopamine injected into mice induced BBB disruption, as demonstrated by the increased extravasation of exogenous dextran and blood-derived leukocytes. Furthermore, specific depletion of Smo in endothelial cells, using a *Tie2-Cre*;*Smofl/fl* mouse line significantly increased BBB permeability [110].

In GBM, the BBB is disorganized, displaying alterations in ECM, tight junctions and basement membrane. The water channel aquaporin-4 (AQP-4), which is specifically localized at the astrocytic endfoot membranes in physiological BBB, is upregulated in GBM and redistributed over the cellular surface. Upregulation of AQP4 in GBM is associated with loss of Agrin, a component of the ECM. Accordingly, in *Agrin*-deficient mice, the BBB is intact but AQP4 is no longer restricted vessel-directed membrane domains [111, 112].

Modeling the blood–tumor barrier is important for delivery of therapeutic substances to malignant brain tumors. It is believed that 98 % of small-molecule drugs do not cross the BBB [113]. For a small-molecule drug to cross the BBB in pharmacologically significant amounts, the drug must fit the dual molecular characteristics for lipid-mediated free diffusion across the BBB: molecular mass <400-Da and high lipid solubility [113]. Drugs that fulfill these criteria may still not be able to reach therapeutic levels in the CNS, as they may be substrates of the efflux transporters at the BBB [114].

Although there are numerous GEMMs that have been used to study the formation and biology of BBB under physiological conditions, to our knowledge there is no reported GEMM that investigates the biology of the BBB in the tumoral context. Agarwal and colleagues recently used a xenograft glioma mouse model to study the action of the BBB on the brain distribution of Erlotinib, a small molecule EGFR inhibitor. These authors found that co-treatment of tumor-bearing mice with Erlotinib and pharmacological inhibitors of the BBB efflux transporters P-gp and Bcrp, increased Erlotinib concentration in the brain parenchyma [114].

It is not known whether and how tumor cells contribute to BBB permeability in the context of a brain tumor. There is a need for GEMMs modeling the blood–tumor barrier to understand the contribution of astrocytes and tumor cells to the dysregulation of the BBB.

## Role of GEMMs as preclinical models

Preclinical trials have been mostly performed using mouse xenografts of human brain tumor cell lines. However, these models did not translate into successful results in subsequent clinical trials, probably due to fundamental differences between cell-line derived models and patients’ tumors (see the section on "In vivo approaches for modeling brain tumors").

GEMMs combined to non-invasive imaging techniques (i.e. MRI, bioluminescence) that allow monitoring of tumor development longitudinally [115], are starting to be used for testing targeted therapies. An example is the Rosa26 ODD-Luciferase mouse that can be used to monitor tissue hypoxia in vivo [116]. Since solid tumors often display hypoxic regions, this mouse line can be used to monitor spontaneous tumor development, as has been shown for mammary gland tumors [117]. In another study, Sonabend et al. developed a proneural GBM mouse model by injecting PDGF-IRES-Cre retrovirus into the subcortical white matter of adult mice bearing the floxed tumor suppressors *p53* and *Pten* and a luciferase reporter preceded by a floxed transcriptional stop. The addition of the Cre recombinase in this system inactivates *p53 *and *Pten* and simultaneously activates the luciferase reporter. Bioluminescence monitoring of tumors *in vivo* allowed for therapy benefit evaluation in this mouse model [118].

Recently, a comprehensive in vitro and in vivo high-throughput screen used a mouse model of the *Ephb2*-amplified ependymoma subgroup to identify potential therapies with predicted toxicity against normal NSCs [119]. Importantly, this study found kinases of the insulin growth factor (IGF) signaling and centrosome cycle pathways as regulators of this subtype of ependymoma. Furthermore, this screening model was used to evaluate the activity of the Food and Drug Administration (FDA)-approved anticancer drug 5-fluorouracil (5-FU) against ependymoma cells. Intravenous injection of 5-FU prolonged the survival of tumor-bearing mice, with minimal toxicity against normal NSCs.

In medulloblastoma, the *Ptc1* ^+*/*−^ *p53*-null mouse model was used to assess the activity of a small molecule inhibitor of the Shh pathway. This resulted in reduced tumor growth and increased tumor-free survival [120]. These findings were translated into a clinical trial, in which treatment with the SMO inhibitor GDC-0449 resulted in rapid, although transient, regression of the tumor [121]. However, the tumor recurred and analysis of the patient’s tumor cells revealed the presence, in addition to the initial *PTCH1* mutation, of a mutant *SMO* (*SMO-D473H*) [122]. This mutant was shown to be insensitive to inhibition by GDC-0449. Mouse models are now offering ways to explore the mechanisms of resistance to this SMO inhibitor. A GDC-0449-resistant mouse model was created, in which *Ptc1* ^+*/*−^ *p53*-null mice intermittently treated with GDC-0449 eventually stopped responding to the drug [122]. Interestingly, the *SMO-D473H* mutation identified in the patient was found in one cell line derived from this model. However, in two other cell lines, *SMO* mutations were not detected, suggesting the existence of additional mechanisms to GDC-0449 resistance.

GEMMs are key to understand the mechanisms of tumor progression and dissemination. In the sleeping beauty-driven medulloblastoma model, addition of SB transposition to the *Ptc1* model results in metastatic dissemination through the cerebrospinal fluid system, similar to the pattern seen in children [22]. The patterns of genomic alterations and DNA methylation in this model were confirmed in human tumor-metastases pairs, and further revealed that metastases shared similarities with each other, but were distinct from the primary tumor. These findings suggested that metastases evolve from rare populations of cells in the primary tumors. Signaling pathways that are enriched in both primary tumor and metastases may represent promising therapeutical targets. Targeting the insulin signaling pathway, the most significantly deregulated in metastases, may be a promising strategy, because it is also involved in primary MB tumors [22]. PI3K has been proposed to contribute to the resistance of MB to Shh inhibitors. Therefore, combined inhibition of Shh and Akt/PI3K pathways may represent a promising therapeutical opportunity.

It is important to note that not all primary MB respond to treatment with Shh inhibitors, in agreement with the existence of distinct molecular subgroups. Two independent studies showed that a Myc-driven mouse model of MB does not respond to Shh inhibitors [30, 31]. Moreover, Pei et al. showed that small molecule inhibitors of the PI3K/mTOR pathway inhibited tumor cell growth in vitro and extended survival of tumor-bearing mice, identifying this signaling pathway as a potential therapeutic target for this MB subgroup.

In pediatric gliomas, we recently described the generation of a high-grade astrocytoma model that recapitulates the genetics of a subset of tumors with mutated *BRAF* ^V600E^ and *CDKN2A* deletion. We found that treatment with a combination of the BRAF^V600E^ inhibitor PLX4720 and the CDK4/6 inhibitor PD0332991 had synergistic activity against intracranial tumors in vivo [123]. We observed a similar effect in mouse xenografts of genetically relevant human glioma cell lines. Because these inhibitors yield encouraging clinical results in other cancers, our findings indicate a rational therapeutic strategy for treating a subset of pediatric astrocytomas with *BRAF* ^V600E^ mutation and *CDKN2A* deficiency.

In adult gliomas, a few GEMMs have been used as preclinical models. In a PDGF-driven glioma model, Momota et al. [124] showed that the combination of the chemotherapeutic agent temozolomide with the Akt inhibitor perifosine was more effective than temozolomide treatment alone. This therapeutic strategy may be relevant for the proneural GBM subgroup that harbors the highest number of PDGFRα amplifications [42, 43]. More recently, Zhu et al. [125] used an EGFR-driven GEMM of GBM to test the action of a small molecule inhibitor of the molecular chaperone Hsp90 that promotes cell proliferation. Treated mice displayed increased survival without toxicity. Because Hsp90 inhibition targets multiple signaling pathways, such as EGFR, AKT, CDK4 and CyclinD1, it represents an attractive therapeutic candidate for the treatment of GBMs.

Recently, Chen et al. [126] have created a GEMM to investigate the cellular mechanisms of glioma recurrence. By breeding *hGFAP-Cre*;*Nf1* ^+*/*−^;p53 ^fl*/*fl^;*Pten* ^*fl/+*^ glioma-prone mice to a *Nestin-∆Thymidine kinase-GFP* transgenic mouse line, in which GFP+ NSCs and glioma stem cells (GSCs) can be specifically ablated by ganciclovir, they showed that survival is prolonged upon ganciclovir treatment. In this model, treatment with temozolomide, the first line chemotherapy in GBM, ablated proliferating cells but not quiescent GSCs. This treatment resulted in tumor recurrence. Conversely, combining temozolomide and ganciclovir eliminated both proliferating cells and GSCs, abrogating tumor formation. This study provides the first in vivo evidence of the existence of GSCs, which are responsible for tumor re-growth after treatment. It also highlights the benefits of combination therapies that target different cellular compartments and functions. Recently, Sarkar et al. [127] demonstrated that microglia activation by the FDA-approved drug Amphotericin B (AmpB) reduces GSC tumorigenicity by preventing their proliferation and promoting their differentiation. AmpB treatment of mice bearing xenogeneic or syngeneic GSCs extended survival without substantial toxicity. Importantly, monocytes and macrophages from GBM patients were unable to reduce GSC sphere formation unless they were exposed to AmpB. This observation was confirmed with a case report, where a high-grade astrocytoma patient treated with AmpB for a fungal infection went into apparent remission [127]. These data suggest that AmpB has the potential of treating gliomas and open encouraging new approaches to glioma therapy.

All together, these preclinical studies have provided key aspects on our understanding of tumor development and progression. Studies from Chen et al. and Sarkar et al., demonstrating glioma maintenance by GSC and their targeting by AmpB, and from Wu et al. demonstrating divergence of metastases from primary medulloblastoma and their resistance to therapeutics against the primary tumor, have important clinical implications [22, 126]. They explain why generalized cancer treatments fail, and open new therapeutic avenues to target distinct cellular populations. Tumor subtypes do not respond to the same treatments, therefore, there is a real need to use genetically relevant models during preclinical drug development [30, 31, 123]. However, although the frequency of animals acquiring tumors in GEMMs of brain tumors is high in general (see Table 2), a model with a low tumor frequency would not be suited for preclinical testing, due to the lack of animals.

## Limitations of GEMMs

In the previous sections, we have described how GEMMs can provide critical insights into tumor formation and be used as preclinical models. However, there are several disadvantages to consider when using GEMMs. Distinct genetic background of mouse strains can modify tumor onset and spectrum, complicating the interpretation of a mutation’s effects [128]. For instance, mice heterozygous for *Nf1* and *p53* develop brain gliomas at a higher frequency and grade on the C57Bl/6J background compared to the 129S4/SvJae background [129]. Therefore, the genetic background may affect gene function and tumor susceptibility. Because GEMMs are often analyzed on one genetic background, caution should be exerted when extrapolating results from mice to humans.

Fundamental differences between mouse and human cells may affect tumor development [130]. For instance, there are differences between mouse and human immune systems, including components of the T cell and B cell signaling pathways, cytokine and chemokine expression [131]. Moreover, the basal metabolic rate is higher in mice and humans, and rodents diverge in the spectrum of carcinogens they are susceptible to [130]. In addition, murine cells have active telomerase, whereas telomerase expression is repressed in most mature cells in humans—but activated in embryonic and adult stem cells. In addition, mice have longer telomeres than humans [132]. All these biological differences may have an impact on tumor development.

Importantly, the tumor spectrum in mice may be different from human pathology. Mice tend to spontaneously develop sarcomas (tumors of mesodermal origin), whereas humans are more prone to carcinomas (epithelial tumors) [130]. Telomere dynamics may explain this difference, as shown by the combined *telomerase*- and *p53*-deficient mice that display a shift in the tumor spectrum towards epithelial carcinomas [133]. Some GEMMs of tumor suppressor genes often display a tumor spectrum differing from the human pathology. In humans, inheritance of one mutant *RB* allele predisposes to retinoblastoma but also to osteosarcomas later in life. In contrast, mice hemizygous for *Rb* develop pituitary gland tumors but no retinoblastoma. Retinoblastomas develop in these mice only when a related Rb family member, p107, is deleted [134]. In addition, tumor development in mouse may be different from tumor development in human, due to differences in transcriptional networks. For example, we recently found that the rapid evolution of tandem repeated sequences containing p53 response elements might shape differences in p53 transcriptional networks among mammalian species. The characterization of species-specific p53 target genes may provide a key to improve human cancer modeling in mice [135].

Brain development and organization are globally similar between humans and mice. However, there are anatomical and cellular differences. Beside the presence of convolutions (gyrencephalic brain) in the human cerebral cortex that are absent in the rodent brain, some brain areas show distinct architecture. For instance, in the adult mouse brain, the SVZ harbors NSCs that are tightly associated with ependymal cells, which give rise to transit-amplifying cells; in turn, these cells generate neuroblasts that migrate to the olfactory bulb [24]. In contrast, the human SVZ displays a large band of astrocytes with stem cell properties, separated from the ependymal by a hypocellular gap region [136]. In human brain specimens, neuroblasts migrating from the SVZ are not always observed in the adult [136, 137], but have been detected in infants, where the structure of the SVZ resembles that of the mouse [138]. Other brain cells also show specific functional characteristics in humans. Protoplasmic astrocytes in the human brain are larger and extend more processes than in mice. In addition, human astrocytes propagate calcium signals several fold faster than do rodent astrocytes [139]. Furthermore, the human neocortex also harbors several subclasses of astrocytes, such as interlaminar astrocytes, whose function is unknown and that are not represented in rodents [139]. Whether these species-specific aspects may affect tumor development needs further investigation.

Finally, current models do not recapitulate the intratumoral heterogeneity seen in patients. A recent study used single-cell RNA sequencing to profile individual cells from the same GBM tumor [140]. The authors observed extensive intratumoral heterogeneity at the transcriptional level, including mosaic expression and/or mutational status of RTKs and other signaling molecules. Furthermore, they found that individual tumors contain a spectrum of glioblastoma subtypes [140]. Creating GEMMs that model the intratumoral heterogeneity will be the next challenge in experimental neuro-oncology.

## Conclusions

In vivo models provide unique and valuable information regarding the way brain tumors develop. Comprehensive molecular and cellular characterization of GEMMs will lead to the identification of biomarkers for early stages of tumor development. The generation of molecularly defined GEMMs matching human brain tumor subgroups holds encouraging prospects. Nevertheless, differences in mouse genetic background modifiers, as well as possible differences in tumor development and drug response between mice and humans, should be taken into account in the appreciation of drug efficiency. Therefore, it is essential to integrate accurate GEMMs of brain tumors with xenograft models, genomic and developmental studies, to provide powerful platforms for target identification and drug testing.

## Abbreviations

α-KG: α-Ketoglutarate
AmpB: Amphotericin B
BBB: Blood brain barrier
BLI: Bioluminescent imaging
D-2HG: D-2-hydroxyglutarate
ECM: Extracellular matrix
EGFR: Epidermal growth factor receptor
ENU: *N*-ethyl-*N*-nitrosourea
EP: Ependymoma
FDA: Food and drug administration (USA)
GEMM: Genetically engineered mouse models
GBM: Glioblastoma
GSC: Glioma stem cells
HGA: High grade astrocytoma
IDH1: Isocitrate dehydrogenase 1
IGF: Insulin growth factor
MADM: Mosaic analysis with double markers
MB: Medulloblastoma
MHC: Major histocompatibility complex
MNU: *N*-methylnitrosourea
MRI: Magnetic resonance imaging
NSC: Neural stem cell
NPC: Neural progenitor cell
OPC: Oligodendrocyte precursor cell
PA: Pilocytic astrocytomas
PI3K: Phosphatidylinositide 3-kinase
PDGF: Platelet-derived growth factor
PDGFRα: Platelet-derived growth factor receptor alpha
SB: Sleeping Beauty transposon
SVZ: Subventricular zone
shRNA: Short hairpin RNA
TERT: Telomerase reverse transcriptase
WHO: World health organization

## References

[1] Dolecek TA, Propp JM, Stroup NE, Kruchko C (2012). CBTRUS statistical report: primary brain and central nervous system tumors diagnosed in the United States in 2005–2009. Neuro Oncol.

[2] Northcott PA, Korshunov A, Pfister SM, Taylor MD (2012). The clinical implications of medulloblastoma subgroups. Nat Rev Neurol.

[3] Barth RF, Kaur B (2009). Rat brain tumor models in experimental neuro-oncology: the C6, 9L, T9, RG2, F98, BT4C, RT-2 and CNS-1 gliomas. J Neurooncol.

[4] Lee J, Kotliarova S, Kotliarov Y, Li A, Su Q (2006). Tumor stem cells derived from glioblastomas cultured in bFGF and EGF more closely mirror the phenotype and genotype of primary tumors than do serum-cultured cell lines. Cancer Cell.

[5] Romer J, Curran T (2005). Targeting medulloblastoma: small-molecule inhibitors of the Sonic Hedgehog pathway as potential cancer therapeutics. Cancer Res.

[6] Huszthy PC, Daphu I, Niclou SP, Stieber D, Nigro JM, et al. (2012) In vivo models of primary brain tumors: pitfalls and perspectives. Neuro Oncol 14:979–993 10.1093/neuonc/nos135 PMC3408261 22679124

[7] Singh SK, Hawkins C, Clarke ID, Squire JA, Bayani J (2004). Identification of human brain tumour initiating cells. Nature.

[8] Venere M, Fine HA, Dirks PB, Rich JN (2011). Cancer stem cells in gliomas: identifying and understanding the apex cell in cancer’s hierarchy. Glia.

[9] Chen R, Nishimura MC, Bumbaca SM, Kharbanda S, Forrest WF (2010). A hierarchy of self-renewing tumor-initiating cell types in glioblastoma. Cancer Cell.

[10] Wang J, Sakariassen PO, Tsinkalovsky O, Immervoll H, Boe SO (2008). CD133 negative glioma cells form tumors in nude rats and give rise to CD133 positive cells. Int J Cancer.

[11] Joo KM, Kim J, Jin J, Kim M, Seol HJ (2013). Patient-specific orthotopic glioblastoma xenograft models recapitulate the histopathology and biology of human glioblastomas in situ. Cell Rep.

[12] Klink B, Miletic H, Stieber D, Huszthy PC, Valenzuela JA (2013). A novel, diffusely infiltrative xenograft model of human anaplastic oligodendroglioma with mutations in FUBP1, CIC, and IDH1. PLoS One.

[13] van Miltenburg MH, Jonkers J (2012). Using genetically engineered mouse models to validate candidate cancer genes and test new therapeutic approaches. Curr Opin Genet Dev.

[14] Cheon DJ, Orsulic S (2011). Mouse models of cancer. Annu Rev Pathol.

[15] Serrano M, Lee H, Chin L, Cordon-Cardo C, Beach D (1996). Role of the INK4a locus in tumor suppression and cell mortality. Cell.

[16] Zong H, Espinosa JS, Su HH, Muzumdar MD, Luo L (2005). Mosaic analysis with double markers in mice. Cell.

[17] Liu C, Sage JC, Miller MR, Verhaak RG, Hippenmeyer S (2011). Mosaic analysis with double markers reveals tumor cell of origin in glioma. Cell.

[18] Orsulic S (2002). An RCAS-TVA-based approach to designer mouse models. Mamm Genome.

[19] Copeland NG, Jenkins NA (2010). Harnessing transposons for cancer gene discovery. Nat Rev Cancer.

[20] Koso H, Takeda H, Yew CC, Ward JM, Nariai N (2012). Transposon mutagenesis identifies genes that transform neural stem cells into glioma-initiating cells. Proc Natl Acad Sci USA.

[21] Bender AM, Collier LS, Rodriguez FJ, Tieu C, Larson JD (2010). Sleeping beauty-mediated somatic mutagenesis implicates CSF1 in the formation of high-grade astrocytomas. Cancer Res.

[22] Wu X, Northcott PA, Dubuc A, Dupuy AJ, Shih DJ (2012). Clonal selection drives genetic divergence of metastatic medulloblastoma. Nature.

[23] Genovesi LA, Ng CG, Davis MJ, Remke M, Taylor MD (2013). Sleeping beauty mutagenesis in a mouse medulloblastoma model defines networks that discriminate between human molecular subgroups. Proc Natl Acad Sci USA.

[24] Kriegstein A, Alvarez-Buylla A (2009). The glial nature of embryonic and adult neural stem cells. Annu Rev Neurosci.

[25] Gilbertson RJ (2011). Mapping cancer origins. Cell.

[26] Schuller U, Heine VM, Mao J, Kho AT, Dillon AK (2008). Acquisition of granule neuron precursor identity is a critical determinant of progenitor cell competence to form Shh-induced medulloblastoma. Cancer Cell.

[27] Yang ZJ, Ellis T, Markant SL, Read TA, Kessler JD (2008). Medulloblastoma can be initiated by deletion of patched in lineage-restricted progenitors or stem cells. Cancer Cell.

[28] Li P, Du F, Yuelling LW, Lin T, Muradimova RE (2013). A population of nestin-expressing progenitors in the cerebellum exhibits increased tumorigenicity. Nat Neurosci.

[29] Gibson P, Tong Y, Robinson G, Thompson MC, Currle DS (2010). Subtypes of medulloblastoma have distinct developmental origins. Nature.

[30] Kawauchi D, Robinson G, Uziel T, Gibson P, Rehg J (2012). A mouse model of the most aggressive subgroup of human medulloblastoma. Cancer Cell.

[31] Pei Y, Moore CE, Wang J, Tewari AK, Eroshkin A (2012). An animal model of MYC-driven medulloblastoma. Cancer Cell.

[32] Witt H, Korshunov A, Pfister SM, Milde T (2012). Molecular approaches to ependymoma: the next step(s). Curr Opin Neurol.

[33] Witt H, Mack SC, Ryzhova M, Bender S, Sill M (2011). Delineation of two clinically and molecularly distinct subgroups of posterior fossa ependymoma. Cancer Cell.

[34] Modena P, Lualdi E, Facchinetti F, Veltman J, Reid JF (2006). Identification of tumor-specific molecular signatures in intracranial ependymoma and association with clinical characteristics. J Clin Oncol.

[35] Taylor MD, Poppleton H, Fuller C, Su X, Liu Y (2005). Radial glia cells are candidate stem cells of ependymoma. Cancer Cell.

[36] Johnson RA, Wright KD, Poppleton H, Mohankumar KM, Finkelstein D (2010). Cross-species genomics matches driver mutations and cell compartments to model ependymoma. Nature.

[37] Schwartzentruber J, Korshunov A, Liu XY, Jones DT, Pfaff E (2012). Driver mutations in histone H3.3 and chromatin remodelling genes in paediatric glioblastoma. Nature.

[38] Wu G, Broniscer A, McEachron TA, Lu C, Paugh BS (2012). Somatic histone H3 alterations in pediatric diffuse intrinsic pontine gliomas and non-brainstem glioblastomas. Nat Genet.

[39] Gutmann DH, Parada LF, Silva AJ, Ratner N (2012). Neurofibromatosis type 1: modeling CNS dysfunction. J Neurosci.

[40] da Lee Y, Gianino SM, Gutmann DH (2012). Innate neural stem cell heterogeneity determines the patterning of glioma formation in children. Cancer Cell.

[41] Jacob K, Albrecht S, Sollier C, Faury D, Sader E (2009). Duplication of 7q34 is specific to juvenile pilocytic astrocytomas and a hallmark of cerebellar and optic pathway tumours. Br J Cancer.

[42] The Cancer Genome Atlas Research Network (2008). Comprehensive genomic characterization defines human glioblastoma genes and core pathways. Nature.

[43] Verhaak RG, Hoadley KA, Purdom E, Wang V, Qi Y (2010). Integrated genomic analysis identifies clinically relevant subtypes of glioblastoma characterized by abnormalities in PDGFRA, IDH1, EGFR, and NF1. Cancer Cell.

[44] Brennan C, Momota H, Hambardzumyan D, Ozawa T, Tandon A (2009). Glioblastoma subclasses can be defined by activity among signal transduction pathways and associated genomic alterations. PLoS One.

[45] Phillips HS, Kharbanda S, Chen R, Forrest WF, Soriano RH (2006). Molecular subclasses of high-grade glioma predict prognosis, delineate a pattern of disease progression, and resemble stages in neurogenesis. Cancer Cell.

[46] Noushmehr H, Weisenberger DJ, Diefes K, Phillips HS, Pujara K (2010). Identification of a CpG island methylator phenotype that defines a distinct subgroup of glioma. Cancer Cell.

[47] Sturm D, Witt H, Hovestadt V, Khuong-Quang DA, Jones DT (2012). Hotspot mutations in H3F3A and IDH1 define distinct epigenetic and biological subgroups of glioblastoma. Cancer Cell.

[48] Chen J, McKay RM, Parada LF (2012). Malignant glioma: lessons from genomics, mouse models, and stem cells. Cell.

[49] Jones TS, Holland EC (2011). Animal models for glioma drug discovery. Expert Opin Drug Discov.

[50] Wu X, Northcott PA, Croul S, Taylor MD (2011). Mouse models of medulloblastoma. Chin J Cancer.

[51] Kwon CH, Zhao D, Chen J, Alcantara S, Li Y (2008). Pten haploinsufficiency accelerates formation of high-grade astrocytomas. Cancer Res.

[52] Zhu Y, Guignard F, Zhao D, Liu L, Burns DK (2005). Early inactivation of p53 tumor suppressor gene cooperating with NF1 loss induces malignant astrocytoma. Cancer Cell.

[53] Alcantara Llaguno S, Chen J, Kwon CH, Jackson EL, Li Y (2009). Malignant astrocytomas originate from neural stem/progenitor cells in a somatic tumor suppressor mouse model. Cancer Cell.

[54] Jacques TS, Swales A, Brzozowski MJ, Henriquez NV, Linehan JM (2010). Combinations of genetic mutations in the adult neural stem cell compartment determine brain tumour phenotypes. EMBO J.

[55] Persson AI, Petritsch C, Swartling FJ, Itsara M, Sim FJ (2010). Non-stem cell origin for oligodendroglioma. Cancer Cell.

[56] Lei L, Sonabend AM, Guarnieri P, Soderquist C, Ludwig T (2011). Glioblastoma models reveal the connection between adult glial progenitors and the proneural phenotype. PLoS One.

[57] Friedmann-Morvinski D, Bushong EA, Ke E, Soda Y, Marumoto T (2012). Dedifferentiation of neurons and astrocytes by oncogenes can induce gliomas in mice. Science.

[58] Chow LM, Endersby R, Zhu X, Rankin S, Qu C (2011). Cooperativity within and among Pten, p53, and Rb pathways induces high-grade astrocytoma in adult brain. Cancer Cell.

[59] Song Y, Zhang Q, Kutlu B, Difilippantonio S, Bash R (2013). Evolutionary etiology of high-grade astrocytomas. Proc Natl Acad Sci USA.

[60] Rigau V, Zouaoui S, Mathieu-Daude H, Darlix A, Maran A (2011). French brain tumor database: 5-year histological results on 25 756 cases. Brain Pathol.

[61] Bromberg JE, van den Bent MJ (2009). Oligodendrogliomas: molecular biology and treatment. Oncologist.

[62] Ducray F, Idbaih A, Wang XW, Cheneau C, Labussiere M (2011). Predictive and prognostic factors for gliomas. Expert Rev Anticancer Ther.

[63] Riemenschneider MJ, Jeuken JW, Wesseling P, Reifenberger G (2010). Molecular diagnostics of gliomas: state of the art. Acta Neuropathol.

[64] Yan H, Parsons DW, Jin G, McLendon R, Rasheed BA (2009). IDH1 and IDH2 mutations in gliomas. N Engl J Med.

[65] Dang L, White DW, Gross S, Bennett BD, Bittinger MA (2009). Cancer-associated IDH1 mutations produce 2-hydroxyglutarate. Nature.

[66] Turcan S, Rohle D, Goenka A, Walsh LA, Fang F (2012). IDH1 mutation is sufficient to establish the glioma hypermethylator phenotype. Nature.

[67] Rohle D, Popovici-Muller J, Palaskas N, Turcan S, Grommes C (2013). An inhibitor of mutant IDH1 delays growth and promotes differentiation of glioma cells. Science.

[68] Labussiere M, Idbaih A, Wang XW, Marie Y, Boisselier B (2010). All the 1p19q codeleted gliomas are mutated on IDH1 or IDH2. Neurology.

[69] Killela PJ, Reitman ZJ, Jiao Y, Bettegowda C, Agrawal N (2013). TERT promoter mutations occur frequently in gliomas and a subset of tumors derived from cells with low rates of self-renewal. Proc Natl Acad Sci USA.

[70] Huang FW, Hodis E, Xu MJ, Kryukov GV, Chin L (2013). Highly recurrent TERT promoter mutations in human melanoma. Science.

[71] Hanahan D, Weinberg RA (2011). Hallmarks of cancer: the next generation. Cell.

[72] Bettegowda C, Agrawal N, Jiao Y, Sausen M, Wood LD (2011). Mutations in CIC and FUBP1 contribute to human oligodendroglioma. Science.

[73] Yip S, Butterfield YS, Morozova O, Chittaranjan S, Blough MD (2012). Concurrent CIC mutations, IDH mutations, and 1p/19q loss distinguish oligodendrogliomas from other cancers. J Pathol.

[74] Reifenberger J, Reifenberger G, Ichimura K, Schmidt EE, Wechsler W (1996). Epidermal growth factor receptor expression in oligodendroglial tumors. Am J Pathol.

[75] Robinson S, Cohen M, Prayson R, Ransohoff RM, Tabrizi N (2001). Constitutive expression of growth-related oncogene and its receptor in oligodendrogliomas. Neurosurgery.

[76] Alentorn A, Sanson M, Idbaih A (2012). Oligodendrogliomas: new insights from the genetics and perspectives. Curr Opin Oncol.

[77] Dai C, Celestino JC, Okada Y, Louis DN, Fuller GN (2001). PDGF autocrine stimulation dedifferentiates cultured astrocytes and induces oligodendrogliomas and oligoastrocytomas from neural progenitors and astrocytes in vivo. Genes Dev.

[78] Appolloni I, Calzolari F, Tutucci E, Caviglia S, Terrile M (2009). PDGF-B induces a homogeneous class of oligodendrogliomas from embryonic neural progenitors. Int J Cancer.

[79] Lindberg N, Kastemar M, Olofsson T, Smits A, Uhrbom L (2009). Oligodendrocyte progenitor cells can act as cell of origin for experimental glioma. Oncogene.

[80] Jackson EL, Garcia-Verdugo JM, Gil-Perotin S, Roy M, Quinones-Hinojosa A (2006). PDGFR alpha-positive B cells are neural stem cells in the adult SVZ that form glioma-like growths in response to increased PDGF signaling. Neuron.

[81] Weiss WA, Burns MJ, Hackett C, Aldape K, Hill JR (2003). Genetic determinants of malignancy in a mouse model for oligodendroglioma. Cancer Res.

[82] Sugiarto S, Persson AI, Munoz EG, Waldhuber M, Lamagna C (2011). Asymmetry-defective oligodendrocyte progenitors are glioma precursors. Cancer Cell.

[83] Garraway LA, Sellers WR (2006). Lineage dependency and lineage-survival oncogenes in human cancer. Nat Rev Cancer.

[84] Rowitch DH (2004). Glial specification in the vertebrate neural tube. Nat Rev Neurosci.

[85] Meijer DH, Kane MF, Mehta S, Liu H, Harrington E (2012). Separated at birth? The functional and molecular divergence of OLIG1 and OLIG2. Nat Rev Neurosci.

[86] Ligon KL, Alberta JA, Kho AT, Weiss J, Kwaan MR (2004). The oligodendroglial lineage marker OLIG2 is universally expressed in diffuse gliomas. J Neuropathol Exp Neurol.

[87] Ligon KL, Huillard E, Mehta S, Kesari S, Liu H (2007). Olig2-regulated lineage-restricted pathway controls replication competence in neural stem cells and malignant glioma. Neuron.

[88] Mehta S, Huillard E, Kesari S, Maire CL, Golebiowski D (2011). The central nervous system-restricted transcription factor Olig2 opposes p53 responses to genotoxic damage in neural progenitors and malignant glioma. Cancer Cell.

[89] Sun Y, Meijer DH, Alberta JA, Mehta S, Kane MF (2011). Phosphorylation state of Olig2 regulates proliferation of neural progenitors. Neuron.

[90] Hoshino M (2012). Neuronal subtype specification in the cerebellum and dorsal hindbrain. Dev Growth Differ.

[91] Flora A, Klisch TJ, Schuster G, Zoghbi HY (2009). Deletion of Atoh1 disrupts Sonic Hedgehog signaling in the developing cerebellum and prevents medulloblastoma. Science.

[92] Salsano E, Pollo B, Eoli M, Giordana MT, Finocchiaro G (2004). Expression of MATH1, a marker of cerebellar granule cell progenitors, identifies different medulloblastoma sub-types. Neurosci Lett.

[93] Ayrault O, Zhao H, Zindy F, Qu C, Sherr CJ (2010). Atoh1 inhibits neuronal differentiation and collaborates with Gli1 to generate medulloblastoma-initiating cells. Cancer Res.

[94] Forget A, Bihannic L, Cigna SM, Lefevre C, Remke M et al (2014) Shh signaling protects atoh1 from degradation mediated by the e3 ubiquitin ligase huwe1 in neural precursors Dev Cell 29:649–661 10.1016/j.devcel.2014.05.014 24960692

[95] Charles NA, Holland EC, Gilbertson R, Glass R, Kettenmann H (2011). The brain tumor microenvironment. Glia.

[96] Markovic DS, Vinnakota K, Chirasani S, Synowitz M, Raguet H (2009). Gliomas induce and exploit microglial MT1-MMP expression for tumor expansion. Proc Natl Acad Sci USA.

[97] Zhang J, Sarkar S, Cua R, Zhou Y, Hader W (2012). A dialog between glioma and microglia that promotes tumor invasiveness through the CCL2/CCR2/interleukin-6 axis. Carcinogenesis.

[98] Yeh WL, Lu DY, Liou HC, Fu WM (2012). A forward loop between glioma and microglia: glioma-derived extracellular matrix-activated microglia secrete IL-18 to enhance the migration of glioma cells. J Cell Physiol.

[99] Bajenaru ML, Hernandez MR, Perry A, Zhu Y, Parada LF (2003). Optic nerve glioma in mice requires astrocyte Nf1 gene inactivation and Nf1 brain heterozygosity. Cancer Res.

[100] Thangarajh M, Gutmann DH (2012). Review: low-grade gliomas as neurodevelopmental disorders: insights from mouse models of neurofibromatosis-1. Neuropathol Appl Neurobiol.

[101] Daginakatte GC, Gutmann DH (2007). Neurofibromatosis-1 (Nf1) heterozygous brain microglia elaborate paracrine factors that promote Nf1-deficient astrocyte and glioma growth. Hum Mol Genet.

[102] Bhat KP, Balasubramaniyan V, Vaillant B, Ezhilarasan R, Hummelink K (2013). Mesenchymal differentiation mediated by NF-kappaB promotes radiation resistance in glioblastoma. Cancer Cell.

[103] Assanah M, Lochhead R, Ogden A, Bruce J, Goldman J (2006). Glial progenitors in adult white matter are driven to form malignant gliomas by platelet-derived growth factor-expressing retroviruses. J Neurosci.

[104] Walzlein JH, Synowitz M, Engels B, Markovic DS, Gabrusiewicz K (2008). The antitumorigenic response of neural precursors depends on subventricular proliferation and age. Stem Cells.

[105] Fomchenko EI, Dougherty JD, Helmy KY, Katz AM, Pietras A (2011). Recruited cells can become transformed and overtake PDGF-induced murine gliomas in vivo during tumor progression. PLoS One.

[106] Stock K, Kumar J, Synowitz M, Petrosino S, Imperatore R (2012). Neural precursor cells induce cell death of high-grade astrocytomas through stimulation of TRPV1. Nat Med.

[107] Katz AM, Amankulor NM, Pitter K, Helmy K, Squatrito M (2012). Astrocyte-specific expression patterns associated with the PDGF-induced glioma microenvironment. PLoS One.

[108] Alvarez JI, Katayama T, Prat A (2013). Glial influence on the blood brain barrier. Glia.

[109] Sohet F, Daneman R (2013). Genetic mouse models to study blood-brain barrier development and function. Fluids Barriers CNS.

[110] Alvarez JI, Dodelet-Devillers A, Kebir H, Ifergan I, Fabre PJ (2011). The Hedgehog pathway promotes blood-brain barrier integrity and CNS immune quiescence. Science.

[111] Wolburg H, Noell S, Fallier-Becker P, Mack AF, Wolburg-Buchholz K (2012). The disturbed blood-brain barrier in human glioblastoma. Mol Asp Med.

[112] Rauch SM, Huen K, Miller MC, Chaudry H, Lau M (2011). Changes in brain beta-amyloid deposition and aquaporin 4 levels in response to altered agrin expression in mice. J Neuropathol Exp Neurol.

[113] Pardridge WM (2012). Drug transport across the blood-brain barrier. J Cereb Blood Flow Metab.

[114] Agarwal S, Manchanda P, Vogelbaum MA, Ohlfest JR, Elmquist WF (2013). Function of the blood-brain barrier and restriction of drug delivery to invasive glioma cells: findings in an orthotopic rat xenograft model of glioma. Drug Metab Dispos.

[115] Gross S, Piwnica-Worms D (2005). Spying on cancer: molecular imaging in vivo with genetically encoded reporters. Cancer Cell.

[116] Safran M, Kim WY, O’Connell F, Flippin L, Gunzler V (2006). Mouse model for noninvasive imaging of HIF prolyl hydroxylase activity: assessment of an oral agent that stimulates erythropoietin production. Proc Natl Acad Sci USA.

[117] Goldman SJ, Chen E, Taylor R, Zhang S, Petrosky W (2011). Use of the ODD-luciferase transgene for the non-invasive imaging of spontaneous tumors in mice. PLoS One.

[118] Sonabend AM, Yun J, Lei L, Leung R, Soderquist C (2013). Murine cell line model of proneural glioma for evaluation of anti-tumor therapies. J Neurooncol.

[119] Atkinson JM, Shelat AA, Carcaboso AM, Kranenburg TA, Arnold LA (2011). An integrated in vitro and in vivo high-throughput screen identifies treatment leads for ependymoma. Cancer Cell.

[120] Romer JT, Kimura H, Magdaleno S, Sasai K, Fuller C (2004). Suppression of the Shh pathway using a small molecule inhibitor eliminates medulloblastoma in Ptc1(±)p53(-/-) mice. Cancer Cell.

[121] Rudin CM, Hann CL, Laterra J, Yauch RL, Callahan CA (2009). Treatment of medulloblastoma with hedgehog pathway inhibitor GDC-0449. N Engl J Med.

[122] Yauch RL, Dijkgraaf GJ, Alicke B, Januario T, Ahn CP (2009). Smoothened mutation confers resistance to a Hedgehog pathway inhibitor in medulloblastoma. Science.

[123] Huillard E, Hashizume R, Phillips JJ, Griveau A, Ihrie RA (2012). Cooperative interactions of BRAFV600E kinase and CDKN2A locus deficiency in pediatric malignant astrocytoma as a basis for rational therapy. Proc Natl Acad Sci USA.

[124] Momota H, Nerio E, Holland EC (2005). Perifosine inhibits multiple signaling pathways in glial progenitors and cooperates with temozolomide to arrest cell proliferation in gliomas in vivo. Cancer Res.

[125] Zhu H, Woolfenden S, Bronson RT, Jaffer ZM, Barluenga S (2010). The novel Hsp90 inhibitor NXD30001 induces tumor regression in a genetically engineered mouse model of glioblastoma multiforme. Mol Cancer Ther.

[126] Chen J, Li Y, Yu TS, McKay RM, Burns DK (2012). A restricted cell population propagates glioblastoma growth after chemotherapy. Nature.

[127] Sarkar S, Doring A, Zemp FJ, Silva C, Lun X (2014). Therapeutic activation of macrophages and microglia to suppress brain tumor-initiating cells. Nat Neurosci.

[128] Reilly KM, Loisel DA, Bronson RT, McLaughlin ME, Jacks T (2000). Nf1; Trp53 mutant mice develop glioblastoma with evidence of strain-specific effects. Nat Genet.

[129] Reilly KM, Tuskan RG, Christy E, Loisel DA, Ledger J (2004). Susceptibility to astrocytoma in mice mutant for Nf1 and Trp53 is linked to chromosome 11 and subject to epigenetic effects. Proc Natl Acad Sci USA.

[130] Rangarajan A, Weinberg RA (2003). Opinion: comparative biology of mouse versus human cells: modelling human cancer in mice. Nat Rev Cancer.

[131] Mestas J, Hughes CC (2004). Of mice and not men: differences between mouse and human immunology. J Immunol.

[132] Calado RT, Dumitriu B (2013). Telomere dynamics in mice and humans. Semin Hematol.

[133] Chang S, Khoo C, DePinho RA (2001) Modeling chromosomal instability and epithelial carcinogenesis in the telomerase-deficient mouse. Semin Cancer Biol 11:227–239 10.1006/scbi.2000.0374 11407947

[134] Robanus-Maandag E, Dekker M, van der Valk M, Carrozza ML, Jeanny JC (1998). p107 is a suppressor of retinoblastoma development in pRb-deficient mice. Genes Dev.

[135] Simeonova I, Lejour V, Bardot B, Bouarich-Bourimi R, Morin A (2012). Fuzzy tandem repeats containing p53 response elements may define species-specific p53 target genes. PLoS Genet.

[136] Sanai N, Tramontin AD, Quinones-Hinojosa A, Barbaro NM, Gupta N (2004). Unique astrocyte ribbon in adult human brain contains neural stem cells but lacks chain migration. Nature.

[137] Curtis MA, Kam M, Nannmark U, Anderson MF, Axell MZ (2007). Human neuroblasts migrate to the olfactory bulb via a lateral ventricular extension. Science.

[138] Sanai N, Nguyen T, Ihrie RA, Mirzadeh Z, Tsai HH (2011). Corridors of migrating neurons in the human brain and their decline during infancy. Nature.

[139] Oberheim NA, Takano T, Han X, He W, Lin JH (2009). Uniquely hominid features of adult human astrocytes. J Neurosci.

[140] Patel AP, Tirosh I, Trombetta JJ, Shalek AK, Gillespie SM et al (2014) Single-cell RNA-seq highlights intratumoral heterogeneity in primary glioblastoma. Science 344:1396–1401 10.1126/science.1254257 PMC4123637 24925914

